# Co-Culture of Gut Bacteria and Metabolite Extraction Using Fast Vacuum Filtration and Centrifugation

**DOI:** 10.3390/mps7050074

**Published:** 2024-09-19

**Authors:** Asha Guraka, Richard Duff, Joe Waldron, Gyanendra Tripathi, Ali Kermanizadeh

**Affiliations:** 1 College of Science and Engineering, University of Derby, Derby DE22 1GB, UK; 2 School of Science and Technology, Nottingham Trent University, Nottingham NG1 4BU, UK; gyanendra.tripathi@ntu.ac.uk

**Keywords:** gut bacteria, co-culture, *Bacteriodes thetaiotomicron*, *Limosilactobacillus fermentum*, bacterial metabolites, short-chain fatty acids

## Abstract

This protocol describes a robust method for the extraction of intra and extracellular metabolites of gut bacterial mono and co-cultures. In recent years, the co-culture techniques employed in the field of microbiology have demonstrated significant importance in regard to understanding cell–cell interactions, cross-feeding, and the metabolic interactions between different bacteria, fungi, and microbial consortia which enable the mimicking of complex co-habitant conditions. This protocol highlights a robust reproducible physiologically relevant culture and extraction protocol for the co-culture of gut bacterium. The novel extraction steps are conducted without using quenching and cell disruption through bead-cell methods, freeze–thaw cycles, and sonication, which tend to affect the physical and biochemical properties of intracellular metabolites and secretome. The extraction procedure of inoculated bacterial co-cultures and monocultures use fast vacuum filtration and centrifugation. The extraction methodology is fast, effective, and robust, requiring 4 h to complete.

## 1. Introduction

The human body contains a diverse number of microbes such as bacteria, archaea, fungi, viruses, and other eukaryotes referred to as “microbiota”, with the genomic content of these microbes being referred to as the “microbiome”. The microbiome is complex, with both positive (communalistic, mutualistic) and negative (pathogenic) interactions residing in various anatomical body sites such as the mouth, skin, gastrointestinal tract, and urogenital tract [1,2]. Based on these interactions, it is believed that the human microbiome plays a key role in maintaining metabolic homeostasis and host physiology. This coexisting nature between the host and the microbiome constantly evolves based on both extrinsic and intrinsic factors, such as age, lifestyle, hormonal changes, nutrition, genetic predisposition, environmental conditions, usage of medications, and underlying diseases. The balanced makeup of the microbiome contributes to healthy host physiology, and it is termed as “eubiosis”. On the other hand, any changes from the norm, which is termed as “dysbiosis”, can contribute to the onset of numerous diseases [3,4]. It is important to state that, compared to the other microbial communities of the human body, the majority of microbes reside in the gut. Additionally, it is understood that the gut microbiome has a prominent role in the digestion, metabolism, and regulation of the immune system of the host because it encodes nearly 3.3 million genes that are not encoded in the human genome. The metabolites, proteins, and antimicrobial compounds that are secreted by this gut microbiota showed a potential impact on host health and disease progression [5,6].

As touched upon, over the last decade, numerous studies have reported that changes in gut microbiota might be associated with the onset of a range of metabolic disorders such as obesity, diabetes, and cardiovascular diseases. This highlights the importance of a thorough understanding of the complex metabolic mechanisms between gut microbiota and the host in the development and progression of these diseases [7,8]. However, the key understanding of the molecular mechanisms associated with metabolites and the secretome of gut bacterial populations remains elusive, with more research being required in this area. In microbiology, conventional monocultures are limited in their ability to fully replicate the complex interactions and metabolic diversity found in natural microbial communities. This limitation results in less physiologically relevant metabolic profiling, as fewer diverse chemical interactions and compounds were observed. Alternatively, genetically modified strains can be used to target specific genomic regions to produce pathway-specific metabolites. However, this approach is also time-consuming and often fails to identify the exact compounds involved in understanding disease mechanisms [9,10].

To overcome these limitations, performing bacterial co-cultures has gained significant attention due to their abilities regarding facilitating metabolic induction, cross-feeding, and horizontal gene transfer. In co-cultivation, one species triggers the activation of either the up- or down-regulation of gene clusters of another species, which results in the production of specialised secondary metabolites, antimicrobial compounds, and different analogues within the same gene cluster [9,11,12]. Investigating these complex bacterial interactions is crucial for accurately identifying the compounds involved in disease mechanisms and understanding how microbial communities influence health and disease states. By simulating the natural environment more closely, co-cultures can reveal insights into the metabolic pathways and regulatory networks that are not observable in monocultures or through genetic modifications alone, thereby enhancing our understanding of disease-related metabolic processes [11,12]. Over the years, various co-culture methodologies have been explored, with direct mixing being particularly popular. This method enables microorganisms to interact within the same medium, facilitating the exchange of signalling molecules and metabolites. It has demonstrated enhanced functionality in applications such as bioremediation, bioactive compound production, and fermentation, often outperforming monocultures [13,14,15]. In this protocol, we specifically highlight the direct mixing method between two gut bacteria, showcasing its significance in promoting microbial interactions and optimizing metabolic outcomes.

The extraction of bacterial metabolites is a complex and multi-faceted process. In the past, metabolomes were predominantly extracted using conventional methods, such as quenching with liquid nitrogen and chemical cell lysis using organic solvents [9,16,17,18]. However, several studies have highlighted the specific challenges with these methodologies and the critical need for efficient metabolite extraction [17,19]. Additionally, it has been suggested that traditional rapid and effective cell quenching and extraction techniques, such as filtration-based methods, pose additional challenges, particularly in the context of bacterial cell heterogeneity. Gram-positive species contain rigid cell walls that make it difficult to achieve efficient lysis, potentially leading to incomplete metabolite recovery. Moreover, the use of rapid quenching methods may not fully capture transient metabolic states, and direct solvent interactions lead to cellular disruption, causing loss or degradation of sensitive metabolites and further complicating accurate analysis [17,19].

Given the critical importance of understanding the gut microbial functions and their impact on host health and physiology, Bell et al. discussed the extraction and analysis of small volatile microbiome metabolites in their protocol using human or mouse microbiome samples, such as feces and tissues, by performing homogenisation in extraction solvents and the lyophilisation of samples before analysing using GC-MS. However, the protocol acknowledged limitations such as the instability of volatile metabolites, especially those in low concentrations, as they are prone to evaporation and degradation during the extraction process, making it difficult to capture a comprehensive metabolite profile. In addition, and importantly, species variation poses a significant challenge, where metabolites can vary significantly between human and mouse microbiomes as well as between individual subjects. This variation complicates comparative analysis and can lead to inconsistencies in metabolite identification and quantification [20]. Further compounding these issues, recently Wan et al., highlighted the importance of culturomic strategies in both culture-dependent and culture-independent methods in studying the metabolite interactions of the human gut microbiome. However, culturomics faces challenges such as being labour-intensive and time-consuming, difficulties in recreating specific growth conditions in vitro, and issues with microbial dormancy where certain microbes are difficult to culture under laboratory conditions. This often restricts the range of microbial metabolites that can be studied. Despite its advancements, culturomics still struggles with an incomplete understanding of microbial physiological states and interactions, highlighting the need to address gaps in our understanding of microbial physiology and interactions [21].

To address these challenges, the optimised “fast vacuum extraction” protocol developed in this study provides a controlled and refined approach for metabolite extraction by minimising the exposure of organic solvents and excessive mechanical forces. This reduces the risk of metabolite interactions with harsh conditions leading to degradation and leakage. Furthermore, the protocol enables adaptability across diverse bacterial strains and co-culture environments to address the variability in microbial responses, thereby improving the reproducibility and reliability of the extraction process. This method ensures a comprehensive recovery of the metabolome, preserving the physiochemical integrity of the metabolites and enhancing the overall accuracy and validity of subsequent metabolomic analyses of both monocultures and co-cultures.

In this protocol, we describe the experimental steps of isolation and inoculation of the monocultures and co-cultures of *Bacteriodes thetaiotaomicron* (*B. thetaiotaomicron*) and *Limosilactobacillus fermentum* (*L. fermentum*) from human faecal samples before the employment of a fast vacuum filtration and centrifugation method to isolate a variety of metabolites without affecting the physiochemical and biochemical properties of the bacterial proteins. These bacteria were chosen due to their significant roles in the eubiosis and dysbiosis of metabolic disorders by maintaining gut health and promoting gut barrier activity. *B. thetaiotomicron* is a gut symbiont that plays a crucial role in maintaining gut health by breaking down complex carbohydrates and fibre. It produces most short-chain fatty acids (SCFAs) and aids in promoting gut barrier activity through biofilm aggregates [22,23]. *L. fermentum* is an anaerobic probiotic that promotes gut health and has anti-diabetic properties, such as reducing blood cholesterol levels and improving glycaemic control. It is also known as a heterofermentative bacteria that can metabolise SCFAs and other lactic acids [24,25]. The extraction methods can be utilised for extraction of the entire metabolome and secretome from the bacterial population/s described. Importantly, the extraction protocol can be used for a variety of bacterial species, opening a novel understanding of microbiome/human interactions.

## 2. Experimental Design

### 2.1. Materials for Isolation of Gut Bacteria from Human Faecal Samples

1. Hungate Anaerobic tubes, 16 × 125 mm (Chemglass Life Sciences, Vineland, NJ, USA, Cat. No. CLS-4208-10);
2. AnaeroGen^TM^ 2.5 L sachets (Thermo Scientific, OXOID^TM^, Basingstoke, UK, Cat. No. AN0025A);
3. AnaeroJar, 2.5 L (Thermo Scientific™ AG0025A, OXOID, Basingstoke, UK);
4. Fastidious Anaerobe broth (FAA broth) (E&O Labs, Bonnybridge, UK, Cat. No. KM0188-500G-500);
5. Fastidious anaerobe agar with 7% horse blood (FAA + 7% HB) plates (E&O Labs, Bonnybridge, UK, Cat. No. PP1560-P090);
6. Fastidious anaerobe agar with 7% horse blood and neomycin plates (FAA + 7% HB+ NEO) (E&O Labs, Bonnybridge, UK, Cat. No. PP0140-P090);
7. Fastidious anaerobe agar with 7% horse blood, neomycin, and Aztreonam plates (FAA + 7% HB+ AZT) (E&O Labs, Bonnybridge, UK, Cat. No. PP0144-P090);
8. de Man, Rogosa, and Sharpe (MRS) agar (OXOID, Basingstoke, UK, Cat. No. CM0361);
9. Brain heart infusion (BHI) agar (OXOID, UK, Cat. No. CM1136);
10. Phosphate-buffer saline tablets (Thermo Scientific™, OXOID, Basingstoke, UK Cat. No. 10209252);
11. Sterile L-shaped plastic spreaders (Scientific labs, Nottingham, UK, Cat. No. 222088);
12. Sterile plastic disposable inoculation loops (Fischer Scientific, Horsham, UK, Cat. No. 12870155).

#### Equipment

- Anaerobic chamber (Don Whitley Scientific, Bingley, UK);
- Digital microbiology incubator with shaker (Labnet, Edison, NJ, USA, Cat. No. I-5311-DS);
- Digital Microscope VHX-7000N (Keyence, Milton Keynes, UK).

### 2.2. Materials for Metabolite and Secretome Extractions

1. 99.8% Methanol (HPLC grade, Sigma Aldrich, Dorset, UK, CAS. No. 67-56-1);
2. Acetonitrile (HPLC grade, Sigma Aldrich, Dorset, UK, CAS. No. 75-05-8);
3. Sintered glass funnel and vacuum filter holder kits. 47 mm (Merck, Feltham, UK, Cat. No. 15711719);
4. Buchner filtering Flask (Sigma Aldrich, Dorset, UK, Cat. No. Z740686-6EA);
5. 0.22 µm, 25 mm, hydrophilic membrane filters (Merck, Feltham, UK, Cat. No. GSWP02500);
6. Dry ice;
7. Petri dishes (sterile, Scientific laboratory supplies, Nottingham, UK, SLS2000);
8. 5 mL, 1 mL sterile pipette tips and pipettes;
9. Deionised water;
10. Eppendorf tubes (1.5 mL, scientific laboratory supplies, Nottingham, UK);
11. Sterile falcon tubes (15 mL and 50 mL, scientific laboratory supplies, Nottingham, UK).

#### Equipment

- Class 2 Biological safety cabinet (Monmouth Scientific, Dorset, UK, Cat. No. T1800);
- High pressure portable vacuum pump (KNF N035, 30 L/min flow rate, 13 mbar ultimate vacuum, Sigma Aldrich, Dorset, UK);
- Temperature controlled Centrifuge for falcon tubes (Eppendorf, Hamburg, Germany, Cat. No. 5804 R);
- Temperature controlled Centrifuge for Eppendorf’s (Eppendorf, Hamburg, Germany, Cat. No. 5430 R);
- Gas chromatography (5977 B) mass spectrometer (Agilent 8860, Manchester, UK).

### 2.3. Methods for Sample Collection, Co-Culture, Prior to Extractions

#### 2.3.1. Sample Collection and Ethics

For gut bacteria isolation, fresh human faecal samples are obtained from healthy individuals aged 18–60 with no history of antibiotic use in the last three months. The sample collection was conducted by NHS collaborators (Arden Tissue Bank, University Hospitals Coventry and Warwickshire NHS Trust) under NHS ethical approval. The protocol secured approval from HSE Biosafety and Microbiology Containment (https://www.hse.gov.uk/biosafety/, accessed on 13 April 2024) to work with a class 2 bacterium.

#### 2.3.2. The Isolation and Co-Culture of Gut Bacterium: Direct Mixing Method

- Culture and isolation of gut bacteria

Upon acquisition, 1 g of fresh human faecal sample was homogenised by the rapid pipetting of 9 mL of pre-reduced phosphate-buffered saline (PBS) within an anaerobic chamber (Don Whitley Scientific) to maintain anoxic conditions. Next, serial dilutions were performed and inoculated on specific pre-reduced agar plates, such as fastidious anaerobe agar (FAA) plates, with 7% horse blood (E&O Labs); this agar is enriched with essential growth factors, such as haemin and NAD, which are crucial for the growth of fastidious anaerobes. Additionally, FAA plates supplemented with antibiotics such as neomycin and Aztreonam and 7% horse blood (E&O Labs) were utilised to support clinically significant faecal anaerobic bacteria. These antibiotics selectively suppress facultative anaerobes and aerobes, allowing for the creation of a more challenging culture of obligate anaerobes. Moreover, selective media, such as de Man, Rogosa, and Sharpe (MRS) agar (OXOID) for *Lactobacillus* spp. and brain heart infusion (BHI) agar (OXOID,) for more fastidious bacteria, were employed to enhance bacterial isolation and the growth of significant gut bacteria from the faecal samples

- Identification

**Figure 1:**
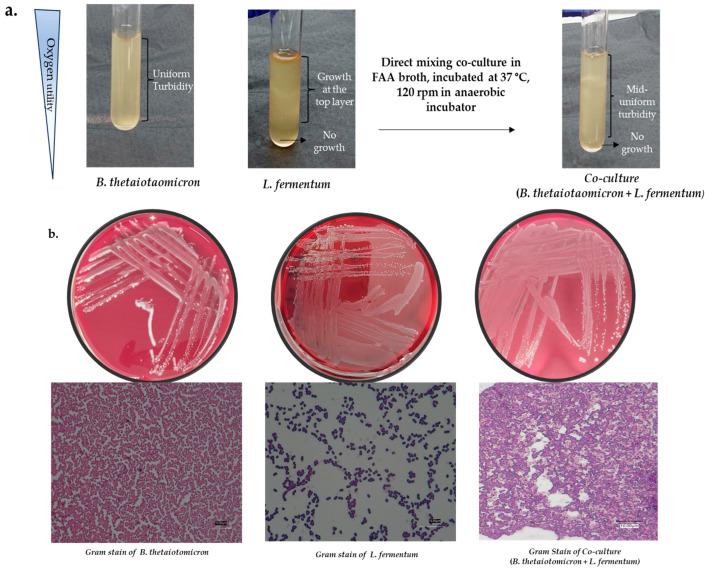

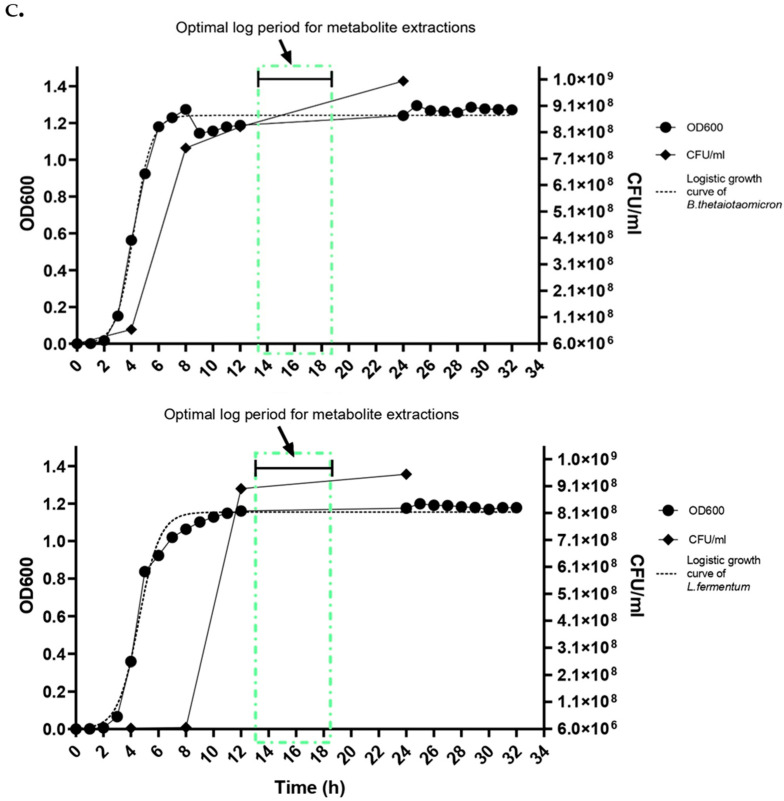
(**a**) Comparison of morphological and distinguishing features of isolated gut bacteria in monocultures and co-cultures: *B. thetaiotaomicron* exhibits uniform turbidity when grown under anaerobic conditions, indicative of its anaerobic nature. *L. fermentum*, a facultative anaerobe, shows turbid growth primarily at the top of the culture medium. In co-culture (1:2 concentrations of *B. thetaiotaomicron* and *L. fermentum*), both uniform and surface turbidity is observed, demonstrating the distinct growth patterns and interactions between the two species. (**b**) Growth and morphological analysis features of *B. thetaiotaomicron* and *L. fermentum* in monoculture and co-culture: *B. thetaiotaomicron* displayed a smooth, cloudy surface and pale beige to dull white colonies, characterised by Gram-negative, non-sporing, and pleomorphic rods. *L. fermentum* manifested as grey, flat, non-cloudy, small colonies characterised by Gram-positive, non-sporing rods or coccoid rods. In co-culture, the distinct growth of both bacteria was observed. Gram staining differentiated the Gram-positive *L. fermentum* (coccoid rods) and Gram-negative *B. thetaoitomicron* (rods). The data showed synergistic interactions between the species with no inhibitions, demonstrating a successful co-culture under optimal growth conditions of FAA + 7% HB media compositions at 37 °C. The Gram-stain images are taken using a digital microscope (Keyence). (**c**) Optimised growth rates of *B. thetaiotaomicron* and *L. fermentum* using OD600 and CFU/mL for metabolite extractions. It is determined between 14 and 18 h using serial dilutions in a 1:2 ratio. Growth curves were analysed using a non-linear regression of logistic growth with a GraphPad Prism 10.

For bacterial identification, colony PCR 16S rRNA using Sanger sequencing was employed using the oligonucleotide group-specific primers mainly to target the V3-V4 hypervariable regions of 16s RNA bacterial genes, which is an important gene marker used in establishing phylogenetic and taxonomic relationships between bacteria, followed by Sanger sequencing for the identification of bacteria [26,27]. The primers included in this study are a universal primer set (Eub338F: ACTCCTACGGGAGGCAGCAG and Eub518R: ATTACCGCGGCTGCTGG), a specific primer set for *Lactobacillus*, *Leuconostoc*, and *Pediococcus* species (F_Lacto05: AGCAGTAGGGAATCTTCCA and R_Lacto04: CGCCACTGGTGTTCYTCCATATA), as well as a primer set targeting *Firmicutes* (Firm934F: GGAGYATGTGGTTTAATTCGAAGCA and Firm1060R: AGCTGACGACAACCATGCAC) [26,27,28]. The sequencing results confirmed the identification of *B. thetaiotaomicron* and *L. fermentum*. The growth conditions for pure monocultures of *B. thetaiotaomicron* and *L. fermentum* were further optimised in fastidious anaerobe broth (FAA) and FAA + 7% HB agar plates at 37 °C for 48 h (Figure 1a,b).

- Co-culture of identified bacterium

To determine identified bacterial concentrations, serial dilutions were performed from 10^−1^ to 10^−6^, with colony enumeration being conducted from the 10^−5^ to 10^−6^ dilutions for both species. Based on these enumerations, the optimal concentration of each bacterium was established, normalizing their ratio to 1:2 of *B. thetaiotaomicron* and *L. fermentum*. Following this normalisation, each bacterium’s growth curve was individually performed to determine the optimal log phase by using optical density at 600 nm (OD600) for 32 h, and colony-forming units per millilitre (CFU/mL) were monitored at 0, 4, 8, 12, and 24 h to ensure the accuracy and reliability of the growth analysis. The choice to measure CFU/mL at these critical time points was intended to validate the OD600 readings, as OD600 can reflect both live and dead cells. Co-culturing of the two bacterial species was then conducted by using the optimised concentration ratios at 37 °C for a minimum of 34 h, ensuring that the cultures reached the mid-log phase of growth, a period characterised by heightened metabolic activity (Figure 1a–c). This phase is critical for maximizing the extraction of intra- and extracellular metabolites, proteins, and enzymes, thereby facilitating a comprehensive analysis of bacterial metabolic outputs.

## 3. Procedure

### 3.1. Day 1

1. Inoculate the isolated monocultures and co-culture (*B. thetaiotaomicron* and *L. fermentum*) in liquid broth FAA media and incubate at 37 °C in anaerobic conditions to reach the mid-log phase with a biomass yield of 10^8^ CFU/mL. This is determined by performing a growth curve to understand the optimal log phase prior to the experiments.
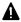
**CRITICAL STEP:** Cultures reaching 10^8^ CFU/mL, OD600 = 0.9 is crucial as it tends to release a higher response in metabolomic profiling.
2. Prepare extraction solvents: 80% Methanol (*v*/*v*) is used for denaturing and solubilizing bacterial molecules and volatile organic compounds. Acetonitrile–Methanol–Water (*v*/*v*) in a 2:2:1 ratio, which dissolve lipophilic substances, hydrophobic substances, and non-polar substances such as amino acids. These solvents should be stored at −20 °C overnight before extractions.
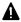
**CRITICAL STEP:** Solvents should ice chilled in −20 °C before and during the experiments to ensure the stability, reproducibility, and reliability of the obtained metabolomes.
3. Required consumables and filtration equipment should be autoclaved.

### 3.2. Day 2

1. Before removing the culture media from −20 °C, set up the filtration equipment by inserting the glass funnel into the vacuum flask, which is attached to the vacuum pump using autoclavable tubing.
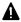
**CRITICAL STEP:** Make sure everything is secure here and place this filtration apparatus in an ice box. This will minimise the loss of metabolites, proteins, and other biochemical compound yields during the procedure.
2. Using forceps, place the 0.22 µm membrane filter paper carefully on the fritted glass base. Next, place the glass funnel on top to cover the porous surface of the membrane filter paper and seal it tightly before the vacuum pump is turned on.
3. Aliquot 3 mL of any extraction solvent mentioned above based on the metabolite of interest in a Petri dish before placing it on dry ice.
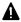
**CRITICAL STEP:** Using 3 mL of solvent during the extractions helps to solubilise highly volatile compounds and the desired metabolites while also helping to characterise them easily.
4. Pipette 15 mL of bacterial culture onto the centre of the membrane filter paper during vacuum pressure.
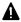
**CRITICAL STEP:** During this step, bacterial cells are filtered without adding any solvents to the culture, including deionised water on the membrane filter paper prior to filtration, to obtain pure extractions.
5. Remove the membrane filter paper from the funnel and place it upside down onto a Petri dish containing extraction solvents.
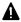
**CRITICAL STEP:** This step is carried out with the new filter papers to ensure extraction is performed with each solvent: (1) 80% Methanol (*v*/*v*) and (2) Acetonitrile–Methanol–Water (2:2:1 *v*/*v*) were used to ensure extraction is made individually.
6. Lyse the bacterial cells by swirling the solvent across the membrane filter before evenly distributing the solvent using a 1 mL pipette 20 times.
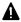
**CRITICAL STEP:** This should be performed as quickly as possible to minimise the amount of time that the culture spends outside of its growth condition, as the cellular metabolism changes rapidly. The speed of step is key to better metabolite recovery and yield.
7. Immediately transfer the solvent with extracted metabolites into 1.5 mL Eppendorf tubes and place them in dry ice until proceeding to the next step.
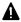
**CRITICAL STEP:** Dry ice helps to maintain the stability of the volatile nature of compounds extracted in the solvent.
8. Repeat steps 5 to 9 for the rest of the bacterial cultures, if required, or discard the culture.
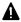
**CRITICAL STEP:** It is advised to repeat the steps in order to maintain the reliability and reproducibility of the experiments.
9. Once the filtration and extraction are completed, Eppendorf tubes are centrifuged at 4 °C maximum speed at 3000 rpm for 5 min.
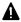
**CRITICAL STEP:** This step is carried out to remove the unwanted cellular debris from the culture and enhance the data analysis of metabolites using OMICS technologies and the quantification of proteins.
10. After centrifugation, collect the supernatant without disrupting the pellet.
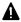
**PAUSE STEP:** After collecting supernatants, store it in −80 °C for long-term storage and used immediately for metabolic profiling and quantification for proteins.
11. Now, to extract the secretome, transfer the filtrate collected in the vacuum flask to individual 15 mL or 50 mL falcon tubes and centrifuge them for 10 min at 4 °C at 1000 rpm maximum speed.
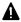
**CRITICAL STEP:** This filtrate is not exposed to any extraction solvents, which will aid in quantifying and gaining a more physiologically relevant understanding of the culture’s secretome analysis.
12. Once centrifuged, collect the supernatant and pellet for the quantification of proteins using BCA assay; can perform Western blots immediately using the pellet as control.
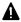
**PAUSE STEP:** Supernatants and pellets are stored separately in −80 °C for long-term usage.
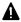
**CRITICAL STEP:** Do not discard the pellet, which can also be used to analyse the influence of co-cultures interactions (positive or negative, i.e., 16S rRNA sequencing. This acts as a control for the secretome analysis. It is advised to use the pellet of 16S sequencing immediately to minimise DNA degradation.

**Figure 2:**
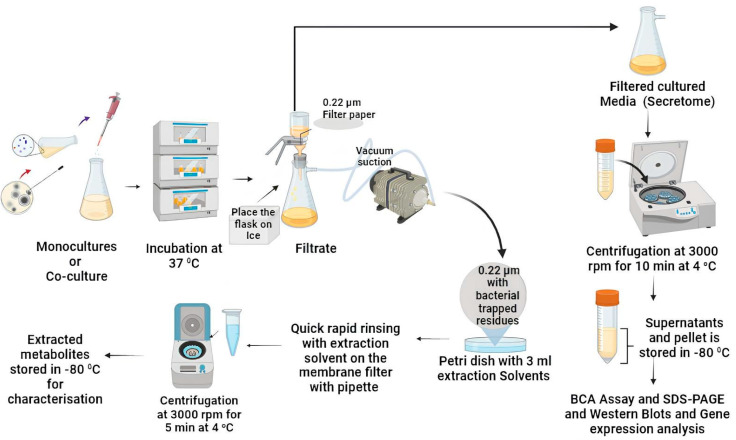
Robust workflow representation of metabolite and secretome extraction employed for the monocultures and co-cultures. Figure created with BioRender.com.

The stesp above has been summarised pictorially in Figure 2.

## 4. Expected Results and Discussion

The objective of this protocol is to extract a comprehensive spectrum of metabolites produced by gut bacteria. This includes short-chain fatty acids (SCFAs) alongside a variety of other crucial biochemical compounds, such as amino acids, lipids, purines, pyrimidines, sugars, and enzyme co-factors. These metabolites are synthesised through the catabolic and anabolic pathways of the gut microbiota, reflecting their metabolic and biological activities and interactions. The identification and quantification can involve advanced metabolomic techniques such as gas chromatography-mass spectrometry (GC-MS), liquid chromatography-mass spectrometry (LC-MS), and/or nuclear magnetic resonance (NMR) [29]. These methodologies allow for the precise quantification and identification of intracellular metabolites and extracellular metabolites, providing a detailed understanding of the microbial metabolic landscape. As an example of the above, an untargeted approach was performed using GC-MS with extracted samples of 80% Methanol (*v*/*v*) as the solvent using the above-described protocol.

**Figure 3:**
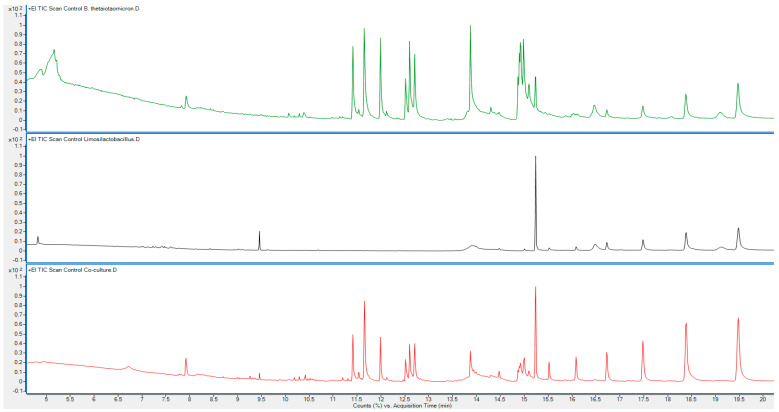
The overlaid chromatograms of three samples are plotted at the percentage of concentration or the intensity amount of analytes vs. retention time for Monoculture 1 *B. thetaiotomicron*; Monoculture 2 *L. fermentum*; and co-culture *(B. thetaiotomicron* + *L. fermentum).* This shows the mass fragmentation of metabolites that are eluted from the three samples.

Since this is an untargeted approach, we have identified a minimum of 20 integrated peaks in all cultures and important gut-related metabolites that are eluted. Using the National Institute of Standards and Technology (NIST) database, the integrated peaks at the same retention times in both monocultures and the co-culture are compared and showed the mass fragments of potential bacterial metabolites (Table 1 and Figure 3).

The above data from the untargeted GC-MS analysis of the metabolite extractions performed using the protocol above demonstrate the presence of a variety of important metabolites, such as short-chain fatty acids (SCFAs), fatty acid amides, diketopiperazines (DKPs), and phenolic compounds among the many biochemical substances. These findings demonstrate that both monoculture and co-culture environments had active metabolic pathways (Table 2). The presence of fatty acid amides such as oleamide and diketopiperazines, as well as SCFAs such as isovaleric acid, butanoic acid, 3-methyl-, and hexanoic acid, 2-methyl-, highlight the effectiveness of the isolation and extraction methods described in this protocol without affecting the physiochemical and biochemical properties of bacterial metabolites across monocultures and co-culture samples that are crucial for gut health, such as those involved in energy production, immune modulation, and microbial interactions.

In conclusion, this protocol allows for the isolation and differentiation of varying bacterial metabolic profiles under controlled environmental conditions. In monoculture setups, individual bacterial species exhibit distinct metabolic signatures that reflect their unique metabolic pathways. Conversely, co-culture experiments reveal metabolic interactions and cross-feeding behaviours among different bacterial species, highlighting the complexity of microbial communities. In regard to the dynamic nature of metabolic interactions in a real gut environment, bacteria are constantly interacting, co-exist, and competing, which underscores the significance of these findings. The protocol’s demonstrated effectiveness suggests that it could be scaled or adapted to study more complex communities. Depending on the specific scientific question, further validation of these findings through performing targeted quantitative analysis of the metabolites is recommended to provide deeper insights.

## Figures and Tables

**Table 1:** Parameters used for untargeted metabolite characterisation of monocultures and co-culture of *B. thetaiotaomicron* and *L. fermentum* using GC-MS.

Parameter	Details
Extraction Solvent	80% Ice cold Methanol
Analytical Technique	Untargeted characterisation using Agilent 8860 gas chromatography (5977B) mass spectrometry with inbuilt NIST database, single quadrupole
GC Column	HP-5MS UI column (30 m × 250 µm × 0.25 μm)
GC Programme	Initial temperature ramp: 50 °C to 280 °C at 50 °C/min; subsequent ramps: 25 °C/min to 200 °C, then 15 °C/min to 280 °C; final hold: 280 °C for 5 min
Total GC Run Time	20.33 min
Carrier Gas	Helium at 1.2 mL/min
Injection Mode	Splitless mode, 1 μL injection volume
Ion Source Temperature	250 °C
Transfer Line Temperature	280 °C
Ionisation Energy	70 eV

**Table 2:** Comparison of eluted metabolites from 80% Methanol extraction solvents of monocultures and co-culture are shown with respective retention times, match factors, and possible pathways linking to gut bacterial metabolism.

Identified Compounds	Monoculture 1 *B. thetaiotaomicron*		Monoculture 2 *L. fermentum*		Coculture		Possible Pathways Linked to Gut Bacterial Metabolism
	RT	MF	RT	MF	RT	MF	
**Isovaleric acid; butanoic acid, 3-methyl-**	4.897	867	**-**	**-**	4.779	750	Short-chain fatty acid (SCFA) metabolism derived from amino acid catabolism [2,10,30,31].
**Hexanoic acid, 2-methyl-**	5.164	810	**-**	**-**	4.961	695	Short-chain fatty acid metabolism (SCFA) [2,30,31].
**Glycerin**	**-**	**-**	**-**	**-**	6.724	619	Central metabolism, glycerol is a play crucial intermediate [32].
**N-methylene-2-phenylethanamine**	7.92	884	**-**	**-**	7.92	912	Amino acid metabolism and secondary metabolite biosynthesis [10,30].
**5-keto-2,2-dimethyl heptanoic acid, ethyl(ester)**	**-**	**-**	**-**	**-**	9.459	579	Fatty acid metabolism and degradation [33].
**Cyclo (L,prolyl-l-valine)**	11.991 12.119	760 641	**-**	**-**	11.991	916	Diketopiperazine metabolism and peptide biosynthesis [34,35,36,37].
**9-Octadecenamide, (Z)- or oleamide**	14.918 14.982	865 792	**-**	**-**	14.918	861	Fatty acid amide metabolism [38,39,40].
**n-heptacosane**	16.456 16.723 17.482 18.379 19.469	707 621 675 775 801	16.082 16.723	790 844	15.003 15.516 16.082 16.723	734 828 843 859	Alkane metabolism, derived from lipid degradation [41].
**Phenol, 2,2′-methylenebis(6-(1,1-dimethylethyl)-4-methyl-**	15.238	826	15.238	909	15.238	922	Phenolic compound metabolism and secondary metabolite biosynthesis [42].

Abbreviations - RT: retention time; MF: match factor.

## Data Availability

Data are available on request.
